# Forward Chemical Genetics in Yeast for Discovery of Chemical Probes Targeting Metabolism

**DOI:** 10.3390/molecules171113098

**Published:** 2012-11-05

**Authors:** Robert St.Onge, Ulrich Schlecht, Curt Scharfe, Marie Evangelista

**Affiliations:** 1 Department of Biochemistry, Stanford Genome Technology Center, Stanford University, Stanford, CA 94305, USA; Email: schlecht@stanford.edu (U.S.); curts@stanford.edu (C.S.); 2 Molecular Diagnostics and Cancer Cell Biology, Genentech, Inc., South San Francisco, CA 94080, USA; Email: evangelista.marie@gene.com

**Keywords:** yeast, forward chemical genetics, chemogenomic profiling, glycolysis, cancer metabolism, Warburg effect, mitochondria, methotrexate, leucovorin

## Abstract

The many virtues that made the yeast *Saccharomyces cerevisiae* a dominant model organism for genetics and molecular biology, are now establishing its role in chemical genetics. Its experimental tractability (*i.e.*, rapid doubling time, simple culture conditions) and the availability of powerful tools for drug-target identification, make yeast an ideal organism for high-throughput phenotypic screening. It may be especially applicable for the discovery of chemical probes targeting highly conserved cellular processes, such as metabolism and bioenergetics, because these probes would likely inhibit the same processes in higher eukaryotes (including man). Importantly, changes in normal cellular metabolism are associated with a variety of diseased states (including neurological disorders and cancer), and exploiting these changes for therapeutic purposes has accordingly gained considerable attention. Here, we review progress and challenges associated with forward chemical genetic screening in yeast. We also discuss evidence supporting these screens as a useful strategy for discovery of new chemical probes and new druggable targets related to cellular metabolism.

## 1. Phenotypic *vs.* Target-based Screening for Drug Discovery

In recent years drug discovery has been dominated by target-based screening, in which large chemical libraries are tested against a single target of interest *in vitro*. Targets are selected based on several factors, including the therapeutic benefit their inhibition is expected to confer, their ability to be assayed in a high-throughput chemical screen, and their “druggability” (whether they possess the structural characteristics necessary to be specifically inhibited by a small-molecule drug). Unfortunately, this approach does not lend itself to the discovery of new druggable targets, and also suffers from the fact that highly potent inhibitors *in vitro*, do not necessarily produce highly selective inhibitors *in vivo*. Many believe the practice of target-based screening has contributed to the dearth of novel small-molecule drugs emerging from the pharmaceutical industry each year despite enormous increases in research and development spending [1,2]. To expand the repertoire of druggable targets, a paradigm shift is needed. Indeed, recent trends suggest a re-emergence of phenotypic screens as the dominant strategy for discovering new, first-in-class, small-molecule therapeutics [3]. Phenotypic screens search for chemicals that elicit a desired cellular phenotype, and the molecular targets of these chemicals are then subsequently identified. Employing cell-based assays for phenotype as the starting point for discovery obviates assumptions made of *in vitro* systems accurately modeling complex intracellular environments. In addition, targets are identified regardless of preconceived notions of their druggability, and therefore phenotypic screens are effective in not only identifying new chemical probes and/or drugs, but also in defining new druggable targets.

## 2. Forward Genetics and Forward Chemical Genetics in Yeast

**Figure 1:**
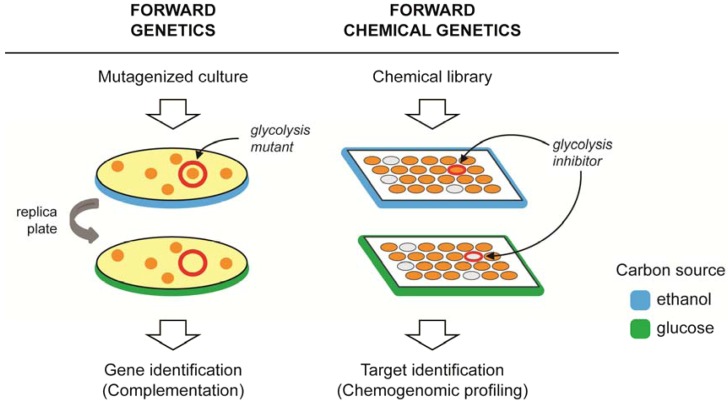
A comparison of forward genetic, and forward chemical genetic screening. Forward genetic screens (**left**) typically involve chemical- or radiation-induced mutagenesis of a wild-type strain, followed by screening mutants for a phenotype of interest, and then identifying the affected gene by complementation. In this example, and as previously described [17], mutants that fail to grow on media containing glucose (green), but not ethanol (blue), identify genes required for glycolysis. In the analogous forward chemical genetic screen (**right**), chemicals that specifically inhibit growth in glucose, but not ethanol, are predicted to identify chemical inhibitors of enzymes in the glycolysis pathway (represented by the wells circled in red).

Few, if any, eukaryotic model systems offer the experimental advantages of the budding yeast, *S. cerevisiae*. Its rapid doubling time and simple growth requirements make it ideally suited for high-throughput phenotypic screening. Though this simple eukaryote cannot fully encapsulate the complexities of a human cell, its contributions to the understanding of core cellular processes in higher eukaryotes have been significant [4,5]. Arguably, its utility has been most defined by forward genetic screens. These screens have identified yeast genes important for cell cycle control, DNA repair, and various metabolic pathways, many of which were subsequently found to be conserved in man [6,7,8,9]. Forward genetic screens typically involve screening a mutagenized culture of yeast for a phenotype of interest, and then identifying the causative genetic locus by complementation (Figure 1, left). For example, the highly conserved DNA repair gene *RAD51*, was identified by first isolating yeast mutants that were sensitive to ionizing radiation-induced DNA damage [10], and then screening for genomic fragments that reversed this sensitivity [11]. 

Analogously, because specific pharmacological inhibition of a protein product will often mimic the phenotypic effects of a loss-of-function genetic mutation [12], forward “chemical genetic” screens represent an alluring way of identifying chemical probes for a wide variety of gene products [13,14]. In these screens, a diverse library of chemical compounds is used in place of random mutagenesis and upon identification of a compound that elicits the desired phenotype, the protein target must then be identified (Figure 1, right). As with genetic screens, growth under a specified condition often represents a convenient phenotype. The availability of chemogenomic tools (*i.e.*, comprehensive collections of gene deletion mutants [15] and over-expression clones [16]) greatly facilitate the process of target identification in yeast. These tools are discussed in more detail in the subsequent section of this review.

Given its past utility in gene discovery/functional annotation, the emergence of yeast as a cornerstone of phenotypic screening for drug and/or chemical probe discovery is seemingly only a matter of time. Some successful screens have already been reported [18,19], and critical experimental barriers have recently been overcome. For example, advancements in combinatorial chemical synthesis have produced millions of commercially available compounds of sufficient purity and supply for high-throughput screening campaigns [20]. Importantly however, several factors can limit the structural diversity of commerical libraries, which in turn limits their utility in defining new druggable targets [21,22]. These factors include the technical hurdles of organic synthesis, and biases in library design, such as adherence to favorable “drug-like” properties [23]. New approaches in chemical library design and synthesis that place a greater emphasis on chemical diversity, hold tremendous potential for identifying new druggable targets in future phenotypic screens [24].

Several high-throughput assays have been developed that enable testing tens-of-thousands of unique chemical entities for effects on yeast growth. Agar-based assays involve transferring small volumes of chemical in high-density array format onto yeast growing on agar plates and then identifying areas (*i.e.*, “halos”) where growth is inhibited or restored [25,26,27]. Similarly, liquid-based assays can test chemicals in small-volume cultures in microtiter plates, in which growth is continuously monitored using a microplate absorbance reader thereby yielding high-resolution growth curves [28,29].

Importantly, poor cell permeability can be an obstacle for chemical screens in yeast, whose cell wall and elaborate chemical defense mechanisms represent a formidable barrier to many compounds. Indeed, it was recently noted that yeast up-regulate drug efflux pump complexes in response to ~31% of 1246 compounds tested [30]. The yeast genes required for the pleiotropic drug response, including many evolutionarily conserved plasma membrane ATP-binding cassette (ABC) transporters, are well known [31] and strains containing combinatorial deletions in nine [32] and sixteen [33] different multi-drug resistance genes have been constructed. As expected, these strains exhibit elevated sensitivity (*i.e.*, their growth is inhibited) to a wide variety of chemical compounds compared to wild-type controls [32,33]. Employing these or similar strains in forward chemical genetic screens will permit a greater fraction of chemicals to enter and remain in the cell, thus improving screen productivity.

## 3. Target Identification Strategies in Yeast

**Figure 2:**
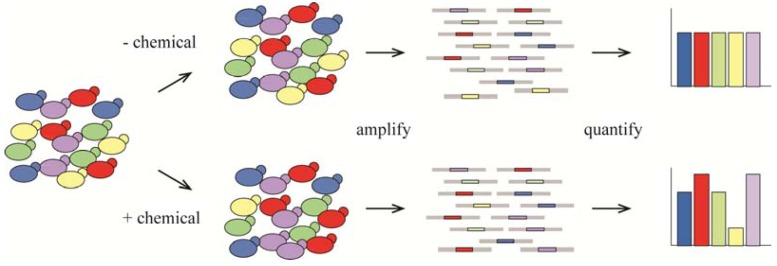
Molecular barcoded yeast collections for characterizing the mechanism of bioactive chemical compounds. A pool of barcoded strains is grown in the presence (bottom) or absence (top) of a chemical inhibitor. Each strain in the pool contains a unique barcode (represented by different colors). Cells are collected, DNA extracted, and barcodes are then PCR amplified using common primers (common primer sites are shown in grey). The individual barcodes in each sample are then quantified, using a tag array or sequencing, and results +/− chemical inhibitor are compared.

The most time-consuming aspect of discovering chemical probes using phenotypic screens is often target identification. A distinct advantage of forward chemical genetic screening in yeast is the availability of powerful chemogenomic assays that facilitate the process of target identification. Following the complete sequencing of the *S. cerevisiae* genome [34], homologous recombination was used to create a complete set of strains harboring precise start-to-stop deletions for each of the ~6,000 yeast genes [15]. These deletion strains are available as haploids (both *MAT*a and *MAT*alpha mating types), as homozygous diploids (in which both copies of a gene are deleted), or as heterozygous diploids (in which one of two gene copies is deleted). In addition, genome-wide collections of yeast ORFs on plasmids were recently constructed and made available to the research community [16]. A key feature of these collections is the inclusion of ‘molecular barcodes’ (20-base oligonucleotides unique to each strain), which allow strains to be combined (*i.e.*, pooled) and assayed in a single tube, thus reducing cost, time, and reagent use. This approach is especially beneficial for studying small-molecules that are costly and/or limited in supply. Because the amount of each DNA barcode reflects the abundance/growth of a strain in the pool, quantification of barcodes using a high-density oligonucleotide array [15,35,36,37], or next-generation sequencing [38,39], allows individual strain fitness to be determined following competitive growth of thousands of strains. Growing these pools in the presence of a chemical inhibitor of growth enables systematic identification of genes that are important for modulating the chemical’s growth-inhibitory effects (Figure 2). These genes could encode direct targets of the inhibitor (see below), or proteins that indirectly affect the inhibitor’s activity. In addition to the *S. cerevisiae* strains described above, molecular barcoding has also been applied for parallel analysis of bacteria [40,41], pathogenic yeast [42,43], and protein-protein interactions [30].

Chemogenomic assays employing collections of yeast gene-deletion mutants and multi-copy clones allow the biochemical target of a chemical to be determined systematically, without any prior knowledge of its mode-of-action (reviewed in [44,45,46]). Many of these assays are based on the principle that for chemical inhibitors of growth, genetic alterations that increase or decrease the abundance of a chemical’s target will confer resistance or sensitivity to that inhibitor, respectively. For example, the power of the heterozygous yeast collection for target identification was first illustrated by Giaever *et al.*, who demonstrated strain-specific chemical sensitivity to compounds acting on the product of the heterozygous locus [47]. The approach successfully identified Alg7 as the target of the natural product tunicamycin [47,48], and leveraging the effects of gene dose on chemical sensitivity has since been used to successfully identify the molecular targets of a wide variety of bioactive compounds in yeast, including many commonly used drugs [49,50,51,52,53,54,55]. An inherent limitation of these fitness-based chemogenomic assays however, is that only targets whose inhibition results in a fitness defect (in either a wild-type or mutant cell) can be identified. For this reason, these assays are perhaps most useful when applied to compounds identified by phenotypic screens based on growth. Still, it should be noted that secondary targets whose inhibition does not produce a growth defect will not be identified by these methods.

The availability of a complete set of gene deletion mutants also facilitates target identification in other ways. Because chemical inhibition of a gene’s protein product is expected to yield similar results as deleting that gene, comparing chemical-induced gene expression changes to transcript levels in deletion mutants, can be used to link a bioactive compound to its cellular target [12]. Similarly, the target of an uncharacterized compound can be identified by comparing its chemogenomic profile, to the chemogenomic profiles of reference compounds for which targets are known [56,57,58]. “Profile matching” can also be applied to genetic interaction data [59], an approach that recently led to the identification of a new chemical inhibitor (dubbed erodoxin) of yeast thiol oxidase (Ero1). Upon comparing the chemical genetic profile of erodoxin to a compendium of genetic interaction profiles, the authors found the genetic interaction profile of *ERO1* to be the best match [60]. In other words, many genes whose deletion resulted in sensitivity to erodoxin, were also synthetic lethal/sick with *ERO1*. The continued expansion of public repositories of genetic interaction data [61] will greatly benefit this method for target identification.

Several other target identification strategies have been successfully applied in yeast. For example, a modified version of the yeast two-hybrid system has demonstrated capability of identifying small-molecule targets [62]. In addition, affinity-capture of proteins binding to immobilized drugs, and their subsequent identification by mass spectrometry (MS), has also proven useful for identifying chemical binding partners in yeast cell extracts [63,64]. These methods however, require covalent modification of the chemical under study, which is not readily amenable to all chemical entities and can have unanticipated consequences on bioactivity. Other MS-based methods that systematically assess potential binding partners in complex cellular extracts, but do not require chemical modification of the inhibitor, have also been described. One such method exploits the stability conferred to a protein upon small-molecule binding and identifies targets as protease-resistant polypeptides in chemical-treated extracts [65]. In another method, targets are identified from proteins that co-fractionate with the chemical following non-denaturing high-performance liquid chromatography [66].

## 4. Metabolism as a Target for Cancer Therapy

It has become increasingly apparent that altered cellular metabolism is a core hallmark of cancer [67,68,69,70]. Since Otto Warburg’s observation that cancer cells ferment glucose even in oxygen-rich environments (*i.e.*, aerobic glycolysis, the so-called “Warburg effect”) a variety of metabolic changes have been linked to cancer progression [71]. This metabolic reprogramming is necessary to support the needs of rapidly dividing tumor cells; namely balancing energy production to support various cellular processes with the *de novo* synthesis of new macromolecules (*i.e.*, proteins, nucleic acids, lipids) to support anabolic growth. Many cancer-causing mutations are now recognized to drive changes in normal metabolic flux and moreover, cellular metabolic state has been linked to clinical outcomes [68,69,72]. For example, Von Hippel-Lindau mutations in renal cell carcinoma drive expression of Hif1 even under normoxic conditions, resulting in glycolytic addiction. Mutations in KRAS and overexpression of Myc regulate a transcriptional program that activates genes governing mitochondrial glutaminolysis resulting in glutamine addiction [73,74]. Directly targeting metabolic changes with small-molecule drugs may be a superior strategy than targeting driver mutations, because it is these changes that underlie the growth and survival capabilities of cancer cells. Furthermore, in contrast to many oncoproteins (for example Myc and KRAS) which are difficult if not impossible to selectively inhibit with small-molecules, metabolic enzymes are generally believed to be a particularly “druggable” class of proteins, because of their innate ability to interact with cellular metabolites. Indeed, the success and wide-spread use of the few existing metabolic drugs (specifically nucleotide biosynthesis inhibitors, *i.e.*, methotrexate, 5-fluorouracil, hydroxyurea, and gemcitabine) support targeting metabolism as a viable avenue for next-generation cancer therapeutics. 

Drugs targeting the altered metabolism of glucose in tumor cells are now actively being pursued [68,75]. Glycolysis inhibitors including 2-deoxy-D-glucose and the putative hexokinase inhibitor lonidamine have been evaluated in several clinical trials. Although these trials were hampered by toxicity to normal tissue, blocking enzymes in the glycolytic pathway remains an attractive therapeutic strategy. Promising glycolytic inhibitors in preclinical or phase I trials include agents that target the glucose transporter GLUT1, phosphofructokinase, tumor-specific pyruvate kinase isozyme M2, and lactate dehydrogenase A [76]. Whether a sufficiently large therapeutic window exists for drugs inhibiting glycolysis remains to be determined, however selective targeting of tumor-specific isoforms of glycolysis enzymes will likely minimize effects on normal cells [77,78]. 

Aerobic glycolysis is a highly inefficient means of ATP production (glycolysis yields two ATP molecules per glucose, whereas oxidative phosphorylation can yield up to 36) and therefore, perhaps not surprisingly, the propensity of tumor cells to exhibit the Warburg effect was initially thought to be due to dysfunctional mitochondria. It’s now apparent however, that most cancer cells harbor mitochondria that are fully able to carry out oxidative phosphorylation, but that instead act primarily as biosynthetic organelles to support the biomass needs of rapidly dividing cancer cells [69]. Drugs that target the altered metabolism of tumor cell mitochondria could promote apoptosis in these cells, or prevent the biosynthesis of key intermediates needed for anabolic growth [79]. Interestingly, metformin, which inhibits complex I of the electron transport chain [80] and which was approved for insulin resistance of type 2 diabetes mellitus, is currently being evaluated in a variety of cancers including ovarian, prostate, pancreatic, colon, melanoma, endometrial, and breast cancer (clinicaltrial.gov). The precise origin of metformin’s therapeutic benefit to cancer patients however, is not entirely understood [68]. 

Other metabolic pathways such as *de novo* fatty acid synthesis and catabolism (β-oxidation) and amino acid biosynthesis and catabolism seem to be just as important as the Warburg effect, if not more so in human cancer. For example, it is now recognized that tumor cells often require high quantities of exogenous amino acids, and specific amino acid auxotrophies in a variety of tumor types have spurred the development of several strategies to reduce glutamine [81], asparagine [82], and arginine [83] in plasma. Other recent discoveries have highlighted the benefits of targeting amino acid biosynthesis pathways directly in tumor cells [84,85]. Inhibiting the *de novo* synthesis of fatty acids [86] has also emerged as an attractive therapeutic strategy, as rapidly dividing cancer cells require fatty acids for the synthesis of new membranes. Compounds inhibiting fatty acid synthesis have shown promise in cancer models and are currently under development [87,88].

## 5. Yeast as a Model for Discovery of Probes Targeting Tumor Metabolism

The realization that altered cellular metabolism is a fundamental enabler of a cancer cell’s ability to grow and thrive has opened a door to new therapeutic opportunities. At the same time, continued development of the yeast model system has facilitated higher-throughput and more effective chemical screens, as well as improved methods for target identification in this organism. Thus, forward chemical genetic screens in yeast for discovering chemical probes directed against conserved metabolic targets, is both highly relevant to human health and extremely timely. 

**Figure 3:**
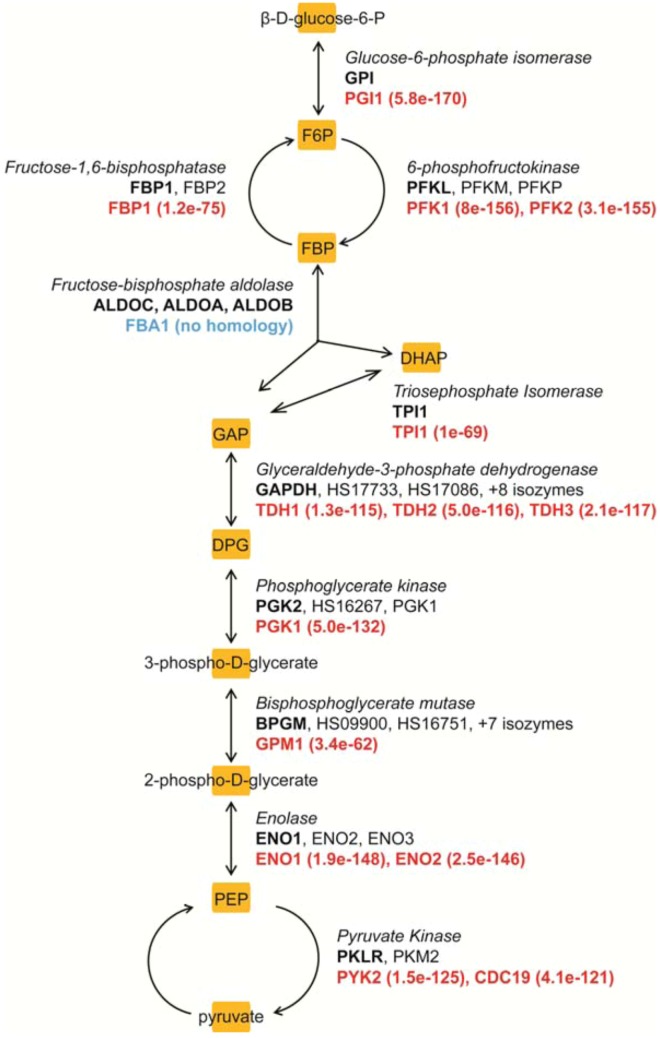
*S. cerevisiae* orthologs of enzymes in the human glycolysis pathway (adapted from http://humancyc.org/). The conversion of glucose 6-phosphate (produced upon phosphorylation of glucose by hexokinase) to pyruvate is illustrated. Metabolites are represented by orange boxes and enzymes catalyzing each reaction are indicated in italics. Human genes encoding these enzymes are indicated in black and homologous yeast genes in red. The yeast fructose-bisphosphate aldolase gene shares no sequence homology with its human counterpart and is indicated in blue. Homology (BLAST E value) between each yeast gene and the human gene in bold is indicated in parentheses. Metabolite abbreviations are as follows; β-D-glucose-6-P = β-D-glucose-6-phosphate, F6P = D-fructose-6-phosphate, FBP = fructose-1,6-bisphosphate, DHAP = dihydroxyacetone phosphate, GAP = D-glyceraldehyde-3-phosphate, DPG = 1,3-bisphospho-D-glycerate, and PEP = phosphoenolpyruvate.

While it is clear that yeast cannot fully represent the complexities of a multicellular organism, core metabolism is highly conserved across eukaryotes [89,90], and thus probes inhibiting yeast metabolic enzymes would likely inhibit the same enzymes in higher eukaryotes. Nearly all yeast enzymes required for the metabolism of glucose to pyruvate contain a great deal of sequence homology with orthologous human enzymes (Figure 3). Similarly, a higher fraction of yeast mitochondrial proteins (60%) exhibit conservation with a human protein, when compared to the entire yeast proteome (46%) [91], which make yeast a particularly useful model for studying mitochondrial function and biogenesis. 

Indeed, the potential of yeast as a model for drug discovery in human mitochondrial disease has recently been demonstrated [27]. The authors used an elegant phenotypic screen in yeast to identify compounds that suppress the respiratory growth defects of a mutant yeast strain. The strain contained mutations that are synonymous to mutations responsible for the NARP (neuropathy, ataxia, and retinitis pigmentosa) syndrome in man, a neurodegenerative disorder caused by abnormalities in mitochondrial energy production. Compounds identified in the screen were active not only in yeast, but also on human cybrid cells derived from NARP patients.

Whether cultured cells rely on glycolysis or oxidative phosphorylation for energy, is often dictated by nutrient availability. For example, HeLa cells (a cervical carcinoma cell line) will primarily utilize glycolysis to generate ATP if glucose is provided in the growth medium, but if instead galactose is provided, ATP is derived primarily from mitochondrial respiration [92,93]. These dependencies were recently leveraged in a phenotypic screen to identify drugs that specifically modulate energy metabolism in human fibroblasts [75]. Similarly, yeast will preferentially ferment glucose for energy (even under aerobic conditions), yet can readily switch to oxidative phosphorylation in the absence of a fermentable carbon source, and thus constitute a compelling cancer model [92,94]. In yeast, non-fermentable carbon sources such as ethanol or glycerol, are metabolized by the tricarboxylic acid cycle independently of glycolysis [95], and therefore, when ethanol or glycerol are used as the sole carbon source in the growth medium, growth depends on 466 nuclear genes that are specifically required for respiration [9]. By extension, inhibitors of these 466 gene products can be thus identified by screening for compounds that specifically inhibit growth in ethanol/glycerol but not glucose-containing growth medium. Conversely, many glycolytic enzymes were first cloned using mutants that grew on ethanol and/or glycerol, but not glucose [17], and thus compounds inhibiting growth in glucose but not ethanol/glycerol-containing growth medium represent potential inhibitors of glycolysis (as illustrated in Figure 1). 

**Figure 4:**
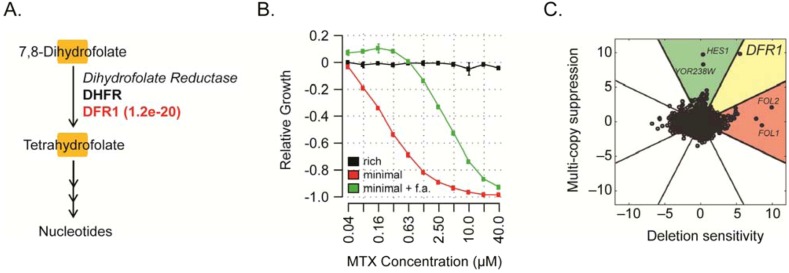
The anti-metabolite cancer drug methotrexate (MTX) inhibits dihydrofolate reductase in yeast. (**A**) Dihydrofolate reductase converts dihydrofolate to tetrahydrofolate. Homology (BLAST E value) between the human enzyme (black) and the yeast enzyme (red) is indicated in parentheses. (**B**) Folinic Acid (*i.e.*, leucovorin) rescues MTX-induced toxicity in yeast. Relative growth (y-axis), the MTX-induced growth inhibition relative to the “no-drug” control, was measured at a variety of MTX concentrations (x-axis) and calculated as described in [30]. Plotted are the mean of three biological replicates. Error bars represent the standard deviation. Relative growth of JHY222 [103] was measured in rich (yeast extract, peptone, glucose) media (black), minimal (yeast nitrogen base plus glucose) media (red), and minimal media plus folinic acid (green). (**C**) Chemogenomic profiling identifies yeast Dfr1 as the target of MTX. MTX-sensitivity of deletion strains is plotted on the x-axis, and MTX-resistance of multi-copy clones on the y-axis. Reproduced with permission from Hoon *et al.* [50].

The few existing cancer drugs that act by targeting metabolism tend to block nucleotide biosynthesis, and it is worth noting that many of these drugs display exquisite mechanistic similarities in yeast [49,50,51,96]. The anti-folate drug methotrexate (MTX) for example, has a long-standing history of success in treating a wide variety of cancers [97], and is often cited as an exemplary cancer drug targeting metabolism. It acts by inhibiting dihydrofolate reductase (DHFR) [98], a key enzyme in folate metabolism and *de novo* nucleotide biosynthesis that catalyzes the reduction of dihydrofolate to tetrahydrofolate (Figure 4A). Consistent with this mechanism of action, MTX-induced inhibition of yeast growth can be alleviated by addition of folinic acid to the growth media (Figure 4B and [99]). Folinic acid (*i.e.*, leucovorin) is readily converted to tetrahydrofolate in the cell (thereby compensating for MTX-induced depletion of this metabolite), and is routinely used clinically to mitigate harmful effects of MTX-based chemotherapy in patients [100]. Notably, MTX has little effect on growth when yeast are cultured in “rich” media (Figure 4B), which provides amino acids, nucleotide precursors, vitamins, and other metabolites that cells would normally synthesize *de novo* [101]. Chemogenomic profiling has revealed that Dfr1 (the yeast orthologue of human DHFR) is indeed the target of MTX in yeast; deleting one copy of *DFR1* in a diploid strain results in elevated MTX-sensitivity, and increasing *DFR1* copy number results in MTX-resistance [49,50,102] (Figure 4C). MTX’s drug-induced auxotrophy (which could be easily detected in a high-throughput phenotypic screen), and its readily identifiable cellular target, underscores the feasibility and potential of forward chemical genetic screens in yeast to identify clinically relevant drug targets and chemical inhibitors. 

A noteworthy limitation of the current yeast chemogenomic toolbox for studying cellular metabolism, are the auxotrophic markers that are present in the barcoded collections described above. The BY4743 background in the yeast deletion collections for example, lacks functional *HIS3*, *LEU2*, and *URA3* genes, thus preventing growth in the absence of exogenous sources of L-histidine, L-leucine, and uracil. These mutations, while convenient for executing standard laboratory protocols requiring selection, ultimately preclude the interrogation of these metabolic pathways with small-molecules. For example, even though 3-amino-1,2,4-triazole (3-AT) is a potent inhibitor of imidazole-glycerol-phosphate dehydratase (His3), this inhibitor has little/no effect on the BY4743 strain because of the *HIS3* deletion and its consequent need for exogenous histidine for survival [12]. Auxotrophic markers may also alter normal flux through key metabolic pathways in less obvious ways. For example, when the *PDA1* gene (encoding a component of the pyruvate-dehydrogenase complex) is deleted in a prototrophic strain, reduced growth on glucose is observed. This phenotype is masked however by the *leu2* auxotrophic marker [104,105]. Therefore, interrogating metabolism with forward chemical genetic screens in yeast should ideally be performed in a prototrophic strain. The recent construction of prototrophic deletion strains for ~4,900 non-essential yeast genes (Amy Caudy, personal communication) will likely prove useful for characterizing chemical inhibitors of metabolic pathways.

## 6. Conclusions and Perspectives

Changes in normal cellular metabolism are required to appropriately balance the energy and biomass needs of rapidly dividing tumor cells, and have thus emerged as a fundamental characteristic of many or all cancers [67,68,69,71]. Exploiting these changes for therapeutic purposes has accordingly gained considerable attention, however, aside from a small number of highly successful examples, few drugs targeting cancer metabolism currently exist. The yeast model system may be particularly useful for the discovery of chemical probes targeting metabolism given the highly conserved nature of metabolic networks, and some noteworthy similarities between yeast and tumor cells (*i.e.*, their common preference to ferment glucose under aerobic conditions [94]). Its experimental tractability and the availability of tools that facilitate drug-target identification, make yeast a convenient organism for high-throughput phenotypic screening. Indeed, the observation that several cancer drugs inhibit yeast and tumor cell growth by identical mechanisms (*i.e.*, the drugs inhibit the yeast ortholog of the human therapeutic target), underscores its potential for discovery of new chemical inhibitors of conserved and medically-relevant targets [49,50,51,96]. Given the large number of potential metabolic targets, as well as the structural diversity of cellular metabolites, it seems likely that future screens will only benefit from advances in diversity-oriented synthesis of new compound libraries [21,22]. Though the inhibitors identified in primary screens will likely require chemical optimization to meet the standards required of a high-quality chemical probe [106], such probes would ultimately provide an important toolbox to improve our technical ability to study metabolism, which remains poorly understood in many cancers [68]. In addition, these probes will likely identify new druggable targets that could inform the search for new medicines, or serve as pre-therapeutic leads themselves.
